# Assessing the effects of different pectins addition on color quality and antioxidant properties of blackberry jam

**DOI:** 10.1186/1752-153X-7-121

**Published:** 2013-07-15

**Authors:** Mariana-Atena Poiana, Melania-Florina Munteanu, Despina-Maria Bordean, Ramona Gligor, Ersilia Alexa

**Affiliations:** 1 Banat’s University of Agricultural Sciences and Veterinary Medicine from Timisoara, Faculty of Food Processing Technology, Calea Aradului 119, 300645, Timisoara, Romania; 2 “Vasile Goldis” Western University of Arad, The Faculty of General Medicine, Pharmacy and Dental Medicine, Feleacului Street 1, 310396, Arad, Romania

**Keywords:** Blackberry jams, Pectin, Monomeric anthocyanins, Polymeric color, Total phenolics, FRAP

## Abstract

**Background:**

In the last years pectin and other hydrocolloids were tested for improving the color stability and the retention of bioactive compounds in gelled fruit-based products. In line with these concerns, our study has been directed to quantify the changes in antioxidant status and color indices of blackberry jam obtained with different types of pectin (degree of esterification: DE, degree of amidation: DA) and doses in response to processing and storage for 1, 3 and 6 months at 20°C.

**Results:**

Blackberry jam was obtained by a traditional procedure used in households or small-scale systems with different commercial pectins (HMP: high-methoxyl pectin, LMP: low-methoxyl pectin and LMAP: low-methoxyl amidated pectin) added to three concentrations (0.3, 0.7 and 1.0%) and investigated in terms of total monomeric anthocyanins (TMA), antioxidant capacity expressed as ferric reducing antioxidant power (FRAP), total phenolics (TP), color density (CD) and percent of polymeric color, PC (%). Thermal processing resulted in significant depreciation of analyzed parameters reported to the corresponding values of fresh fruit as follows: TMA (69-82%), TP (33-55%) and FRAP (18-52%). Biologically active compounds and color were best retained one day post-processing in jams with LMAP followed by samples with LMP and HMP. Storage for 6 months brings along additional dramatic losses reported to the values recorded one day post-processing as follows: TMA (31-56%), TP (29-51%) and FRAP (20-41%). Also, both processing and storage resulted in significant increases in PC (%). The pectin type and dosage are very influential factors for limiting the alterations occurring in response to processing and storage. The best color retention and the highest TMA, TP and FRAP were achieved by LMAP, followed by LMP and HMP. Additionally, a high level of bioactive compounds in jam could be related to a high dose of pectin. LMAP to a level of 1% is the most indicated to provide the highest antioxidant properties in jam.

**Conclusions:**

The retention of bioactive compounds and jam color stability were strongly dependent on the pectin type and dosage. By a proper selection of pectin type and dose could be limited the losses recorded in response to processing and storage.

## Background

Blackberries (*Rubus fructicosus L.*) possess a high level of antioxidant properties which are closely linked to the levels of phenolic compounds such as ellagic acid, tannins, ellagitannins, quercetin, gallic acid, anthocyanins and cyanidins [1-3]. The increasing demand for food with antioxidant action has focused interest on fruit-based products as a good source of biologically active compounds with considerable antioxidant potential. Considering that fresh blackberries are extremely perishable, multiple technologies have been employed to process fresh fruit into various products for long-term preservation [4-6]. Among these products, one of the most popular is jam [7,8]. As a result of health benefits and medical restriction an increasing number of consumers are turning to fruit-based products with low-sugar content due to their high nutritional value [9]. During jam processing, the fruits are subjected to a long heating at high temperature. A significant issue we face during jam processing in households, small-scale or industrial sectors is the negative impact of thermal treatment on the bioactive compounds and consequently, on the antioxidant properties displayed by the obtained products. The studies conducted by Rommel *et al*. [10], Syamaladevi *et al.*[11], Patras *et al.*[12] and Rababah *et al.*[13] have shown that various processing methods of fruits cause serious alterations in their antioxidant properties due to the loss of anthocyanins and phenolic compounds*.* In fruit jams, anthocyanins represent both a source of natural antioxidants and a key parameter for color quality, affecting their acceptance by the consumers [14]. The color deterioration is associated with the loss of anthocyanin pigments or formation of brown pigments [15]. The results reported by Sadilova *et al*. [16] have revealed that during heating, the anthocyanins degradation generally cause the pigments discoloration having a great impact on color quality and also, on their in vitro antioxidant capacity.

The main reason that drove us towards this study was the concern for finding solutions to improve the retention of bioactive compounds in fruit-based products. Recent studies have shown that some hydrocolloids, such as pectin, corn starch, and sodium alginate improve the color stability in gel model systems which were mostly attributed to electrostatic interactions between the positively charged flavylium cations and the dissociated carboxylic groups of the pectin, while other hydrocolloids showed adverse effects or did not show any influence [17]. These studies highlighted the role of non-phenolic food components in stabilizing of anthocyanins in gelled fruit-based products.

Pectin is a high value functional food ingredient widely used as a gelling agent particularly in jellies, jams and spreads. During jam processing, gel formation involves the association of pectin chains that leads to the formation of three-dimensional networks. The ability of pectin to form gel depends on the molecular size and DE [18,19]. The hydrogen bonds that occur between the pectin chains are the main factor responsible in the stabilization of a HMP network. In addition, hydrophobic interactions of the methyl ester groups are essential in gel formation [20]. The gelling mechanism in jam obtained with LMP is based on the clustering of the pectin chains and occurring of some cavities between them as a result of bended shape of the pectin chains. These cavities will be occupied by carboxyl and hydroxyl groups. The formation of these cavities as well as the carboxyl and hydroxyl groups promotes the association of pectin chains by calcium gelation. Therefore, gelling mechanism involves the formation of a continuous network of ionic cross bindings via calcium bridges between the carboxyl groups belonging to two different chains located in close proximity [18,21]. In jam obtained with LMAP, supplementary links by hydrogen bonds occur as a result of the presence of amide groups. In this case, the clustering of pectin chains is more controlled than for LMP, because the network formation is due to the hydrogen bonds between the amid groups and occurs more slowly than the reaction of LMP chains with calcium ions [18,22]. The results of the study performed by Holzwarth *et al.*[23] regarding the influence of different pectins, process and storage conditions on anthocyanins and color of strawberry jams and spreads revealed that low-esterified pectins have proved better stabilizing effects on anthocyanins in fruit-based gelled products than high-esterified pectins. As a result of gel formation based on different types of chain associations, biologically active compounds from fruit jam could be protected against degradation by water attack, condensation reactions or thermal destroying [18,24]. Lewis *et al*. [25] suggested that pectin is involved in the color stabilization of gelled fruit-based products. The study carried out by Poiana *et al*. [26] highlighted the impact of pectin dose on improving the antioxidant properties and color stability of bilberries jam. Also, the results reported by Kopjar *et al.*[24] have revealed the effect of various pectins on the antioxidant activity of raspberry jam. Furthermore, recent studies on this topic conducted by Buchweitz *et al*. [27] and Buchweitz *et al*. [28] have shown the impact of the pectin type on the storage stability of black currant anthocyanin pigments in pectic model solutions.

In line with the current concerns on this topic, the goal of this study was to explore the effects of pectin type (high and low-esterified, amidated) and dosage on color retention and antioxidant properties of blackberry jams after processing and during 6 months of storage at ambient temperature.

## Results and discussion

Blackberry jam obtained with different types of pectin (DE, DA) applied at three concentrations were analyzed one day post-processing (0) and after 1, 3 and 6 months of storage at 20°C in terms of TMA, CD, PC (%), TP and FRAP values. Data resulted from material ratios performed to jam processing showed that to obtain 100 g jam with 45°Bx it was needed approximately 68 g fresh fruit. This information has been used to evaluate the theoretical content of investigated compounds in obtained jams. In Table 1 are shown the main chemical parameters of fresh fruit used for jam preparation. The content of TMA, TP and FRAP values recorded in jam after processing were assimilated with the real values of these parameters. We assumed that the differences between theoretical and real content of measured parameters were due to thermal treatment applied for jam processing.

**Table 1:** Chemical characteristics of fresh blackberries

**Component (Units)**	**Values**
TP (mg gallic acid·100^-1^ g fw)	521.8 ± 12.8
TMA (mg·100^-1^ g fw)	190.1 ± 8.1
FRAP (mM Fe^2+^·100^-1^ g fw)	4.4 ± 0.3
TSS (°Brix)	14.0 ± 0.6
CD	9.9 ± 0.7
PC (%)	6.0 ± 0.4

In order to make more visible the changes of assessed parameters in response to storage, reported to the values recorded one day post-processing (as control), data were processed by one-way ANOVA test. The variation of studied parameters during storage time was presented as star charts which plot the values of each category along a separate axis that starts in the center of the chart and ends on the outer ring. Neighbor-Joining Cluster analysis was performed for clustering of jam samples depending on FRAP values, TMA and TP content to identify the best methods for blackberry jam processing.

### Evaluation of total monomeric anthocyanins content

In Table 2 is presented the TMA content from blackberry jam after processing and storage. Considering the values reported in Tables 1 and 2 can be assessed the losses registered for TMA in response to thermal processing. The first thing we could notice about these data is that TMA content recorded in blackberry jam was much lower than in the corresponding fresh fruit. It is known that anthocyanins exhibit a high sensitivity to temperature [12,14]. Thermal treatments of fruit, especially those involving prolonged exposure at high temperature, cause dramatic alterations of TMA due to oxidation, cleavage of covalent bonds or enhanced oxidation reactions [29]. Thermal processing leads to complexation reactions between anthocyanins and other compounds resulted in response to high temperature exposure. Also, the losses of TMA could be due to formation of anthocyanin polymers or condensation between anthocyanins and procyanidins or other phenolic compounds [5,9,12].

**Table 2:** The impact of storage at 20°C on TMA content of blackberry jam

**Jam samples**	**TMA (mg·100**^**-1**^ **g jam)**			
	**1 day (0)**	**1 month**	**3 months**	**6 months**
LMP1	36.8 ± 0.9	34.1 ± 1.3^ns^	29.6 ± 1.1^**^	22.4 ± 1.0^***^
LMP2	34.0 ± 1.2	31.2 ± 0.9^*^	26.8 ± 1.2^**^	20.0 ± 1.0^***^
LMP3	31.9 ± 0.9	26.9 ± 0.5^*^	23.9 ± 0.8^**^	17.3 ± 0.8^***^
LMAP4	40.7 ± 1.6	38.8 ± 1.3^ns^	34.2 ± 1.3^**^	28.2 ± 0.9^***^
LMAP5	39.1 ± 1.3	36.6 ± 1.2^ns^	32.5 ± 1.4^*^	25.6 ± 1.2^***^
LMAP6	35.8 ± 0.7	31.2 ± 1.1^*^	28.0 ± 1.3^**^	20.9 ± 0.8^***^
HMP7	28.0 ± 0.9	24.7 ± 1.1^*^	20.1 ± 1.1^**^	15.2 ± 0.9^***^
HMP8	26.2 ± 0.9	22.41 ± 0.8^*^	19.8 ± 0.7^**^	13.3 ± 0.8^***^
HMP9	23.3 ± 0.9	19.0 ± 0.9^*^	15.4 ± 0.7^**^	10.2 ± 0.8^***^

Statistical differences are indicated as follows: ns – non-significant, P > 0.1; * – significant, P < 0.05;

** – highly significant, P < 0.01 and *** – extremely significant, P < 0.001.

Legend: LMP1: LMP 1%; LMP2: LMP 0.7%; LMP3: LMP 0.3%; LMAP4: LMAP 1%; LMAP5: LMAP 0.7%; LMAP6: LMAP 0.3%; HMP7: HMP 1%; HMP8: HMP 0.7% and HMP9: HMP 0.3%.

Our data revealed that thermal processing of fresh fruit induced significant losses in anthocyanin pigments, in the range 69-82% from the value registered for corresponding fresh fruit. Our data are consistent with the results of other studies that reported losses in TMA content during jam processing from wild berries in the range 70-85% [7,8,26].

As presented in Table 2, the changes of TMA content in response to thermal processing were affected by the pectin type and dosage. By using of LMAP, LMA and HMP the losses recorded in TMA content were in the ranges: 71-75%, 69-71%, 78-82% reported to the values registered for corresponding fresh fruit. These data revealed that anthocyanin pigments were better retained immediately after processing in samples obtained with low-esterified pectin than in jams with high- esterified pectin. Among the jams obtained with pectins having similar DE, the best retention was noticed by using of amidated pectin. Moreover, the increasing of pectin dose from 0.3 to 1% resulted in improvement of TMA retention in jam. This fact could be explained by interactions between anthocyanins and pectin chains. To support this view we started to the results previously reported by Holzwarth *et al.*[23], Kopjar *et al.*[24] and Buchweitz *et al.*[28] who reported that the pectin type has a great impact on its functionality. The mechanism of gel formation during jam processing is important in explaining of our results. The different types of associations that occur between chains are determined by the pectin type (DE, DA) [17,24,28]. LMP and LMAP probably interact with anthocyanins more easily because they have fewer methoxyl groups than HMP [17,18]. Reporting the previous results to those obtained here, the improving of anthocyanins stability in jam might be explained by the fact that pectins are polyuronic acids and their ability to retain anthocyanins is attributed to electrostatic interactions between the dissociated carboxylic groups of pectin and the flavylium cations of the pigments. The improved stability of pigments in blackberry jam prepared with amidated pectin might be due to the formation of additional hydrogen bonds between the hydroxyl groups of the anthocyanins and the amide groups of pectin [23]. Due to these associations, anthocyanins can be protected against water attack or condensation reactions among anthocyanins and procyanidins [17]. Accordingly, our data are consistent and strengthen the findings developed by Holzwarth *et al.*[23] and Buchweitz *et al.*[28] regarding the effect of pectin type on TMA retention. The aforementioned results might suggest that it is possible to control the content of TMA retained in gelled fruit-based products by pectin type and dose. As presented in Table 2, TMA content significantly decreased during jam storage. Important losses in TMA content during storage of different fruit-based products were also reported by other studies [5,9,15,30]. In the storage time, oxidative reactions occur due to enzymatic activity exhibited by polyphenoloxidase, peroxidase and glucosidase. Moreover, natural light exposure***,*** presence of saccharides and their degradation products will enhance the degree of pigments destruction [31].

**Figure 1:**
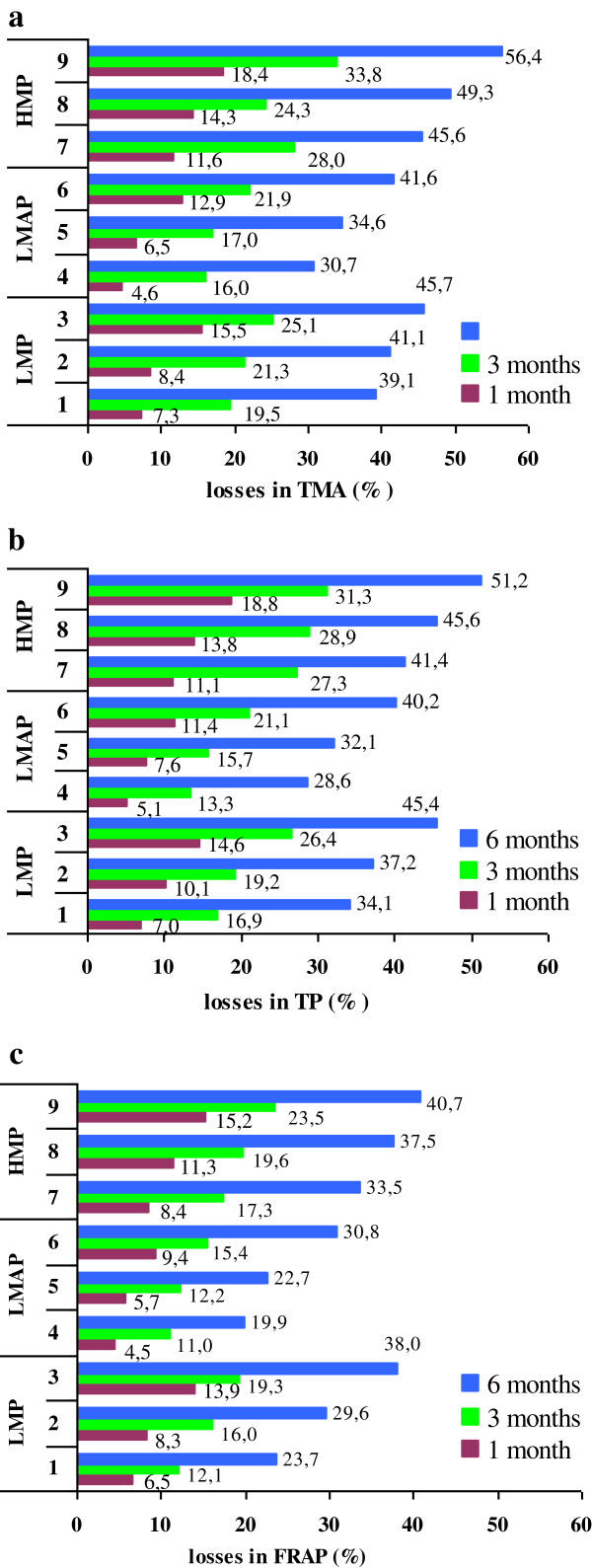
**The relative losses of investigated parameters during jam storage (a: TMA; b: TP; c: FRAP).** Legend: LMP1: LMP 1%; LMP2: LMP 0.7%; LMP3: LMP 0.3%; LMAP4: LMAP 1%; LMAP5: LMAP 0.7%; LMAP6: LMAP 0.3%; HMP7: HMP 1%; HMP8: HMP 0.7% and HMP9: HMP 0.3%.

At the end of storage the relative losses in TMA content were in the range 31-56%, Figure 1a. Interestingly, anthocyanins stability during storage strongly depended on the pectin type and dosage used in jam formulation. After 6 months of storage, the best retention of TMA was noticed in samples with LMAP and the lowest in jams with HMP. Among the jams prepared with low-esterefied pectin, anthocyanins stability was better in samples obtained with pectin having similar DE and amidation, respectively. More researches are needed to study individual anthocyanins to assess if there are any differences in their degradation pattern and stability during jam processing and storage in relation with pectin type and its dosage.

**Figure 2:**
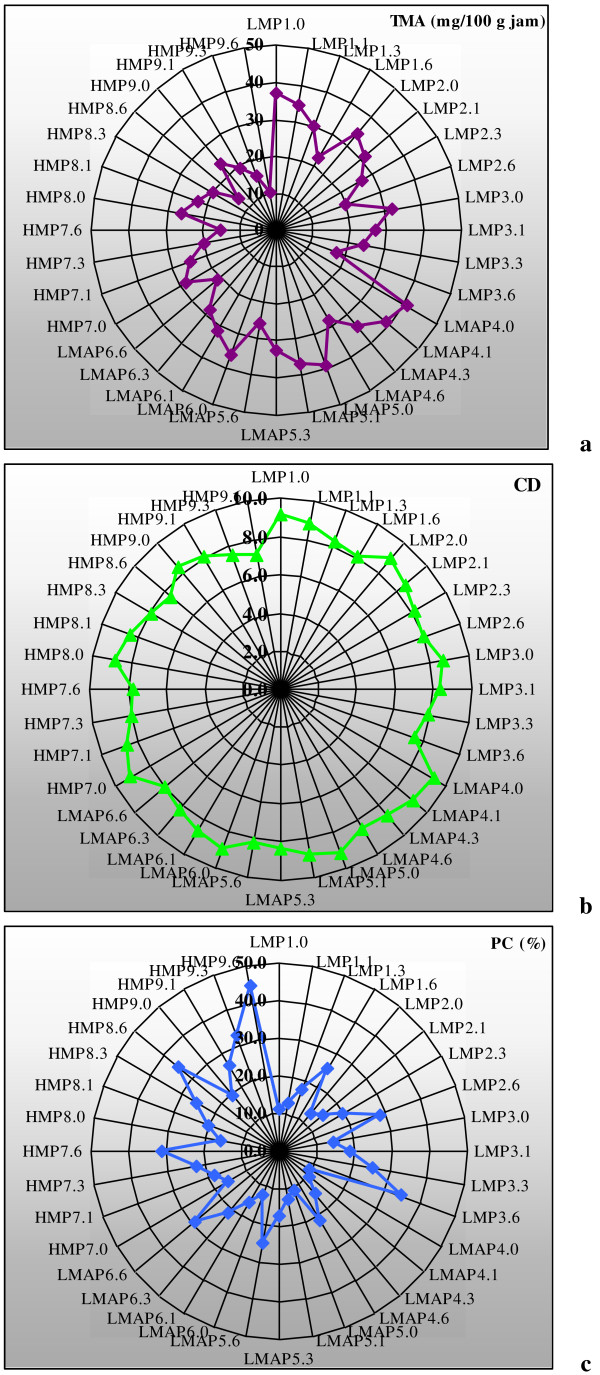
**Star representation of TMA (a), CD (b) and PC % (c) variation during jam storage.** Legend: LMP1: LMP 1%; LMP2: LMP 0.7%; LMP3: LMP 0.3%; LMAP4: LMAP 1%; LMAP5: LMAP 0.7%; LMAP6: LMAP 0.3%; HMP7: HMP 1%; HMP8: HMP 0.7% and HMP9: HMP 0.3%. In samples labeled as LMP1.0, LMP1.6 and so on, the number after point represents the storage time (e.g. LMP1.0: LMP1 one day post-processing; LMP1.6: LMP1 after 6 months of storage).

In Figure 2a is shown the variation of TMA content in response to storage time. It can be noticed that the highest value of TMA corresponds to LMAP4.0 and the lowest to HMP9.6. TMA content, as it appears from Table 2, is closely related to the type and level of pectin used in jam formulation as well as the storage time. From the statistical analysis by ANOVA test it could be noticed that the changes recorded in TMA content was greatly affected by storage period, Table 2. One month of storage induced at most significant differences reported to the control values (P < 0.05) while over 6 months of storage the changes became extremely significant for all jam samples (P < 0.001).

### Evaluation of color indices

**Figure 3:**
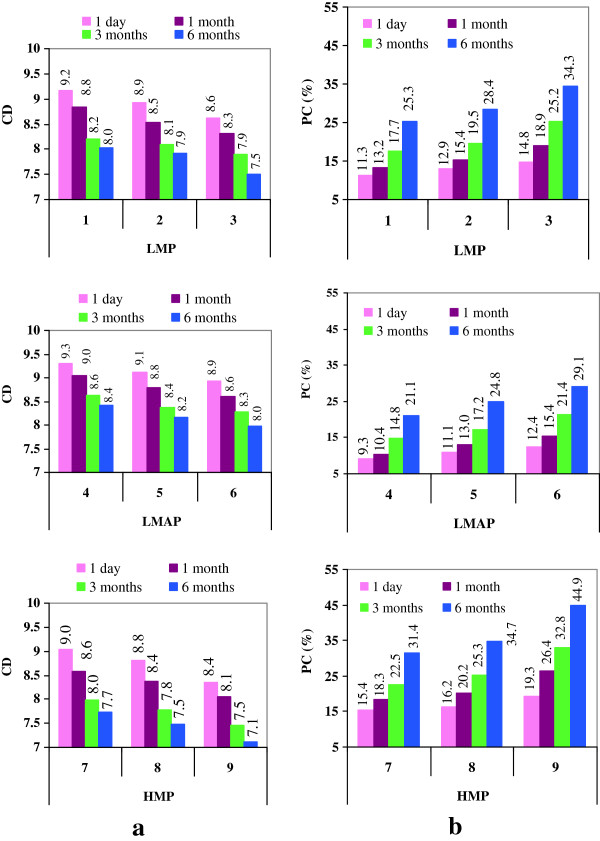
**The impact of storage on the color indices of blackberry jam (a: CD; b: PC %).** Legend: LMP1: LMP 1%; LMP2: LMP 0.7%; LMP3: LMP 0.3%; LMAP4: LMAP 1%; LMAP5: LMAP 0.7%; LMAP6: LMAP 0.3%; HMP7: HMP 1%; HMP8: HMP 0.7% and HMP9: HMP 0.3%.

Figure 3(a,b) provides information on the changes recorded in color of blackberry jam in response to storage. The color quality was quantified by color density (CD) and percent of polymeric color PC (%). It was noticed a certain sensibility of CD to pectin type or dose used for jam preparation. Thus, one day post-processing, jam samples prepared with different types or doses of pectin presented different values of CD in the range 8.4-9.3, Figure 3a. Samples with high-esterified pectin had lower values of CD than samples with low-esterified pectin. Also, it can be seen that jam samples with LMAP had slightly higher values of CD than samples with LMP. This tendency remained during 6 months of storage. After 6 months of storage, the decreases of CD were in the range 10-15% reported to the control. Based on these results, it could be noticed that CD exhibited a good stability in response to long-term storage. The results reported by Mazzaracchio *et al.*[32] suggest that pectin could induce a slight increase in color displayed by flavilium cation that is in equilibrium with the pseudobase at the same pH. In addition, a weak hydrophobic interaction between methoxyl groups of anthocyanin aglycons and methoxyl groups of pectin chains could occur, resulting in a weak co-pigmentation effect [32]. The differences recorded in CD in relation with pectin type might be explain by the fact that low-esterified pectins interact with anthocyanins more easily because they have less methoxyl groups than high-esterified pectins.

Based on star chart, Figure 2b, it can be seen the variation of CD during jam storage. The findings obtained from this chart were in agreement with those revealed by Figure 3a and pointed out that the highest value of CD corresponds to sample LMAP4.0 and the lowest to HMP9.6. Also, the variation registered for CD during jam storage is very low.

PC (%) is a useful parameter for assessing the color quality because provides information regarding the percentage of color represented by polymerized material [10,33]. Thermal processing led to the occurrence of polymeric pigments revealed by increasing of PC (%) from 6.0% (in fresh fruit) to the values in the range 9-19% in jam samples, Figure 3b. Significant increases in PC (%) have also been noticed for other thermally treated, shelf-stable products from blackberries (juices, canned products, and purees) during storage at 25°C [5].

During jam processing fruit are exposed to thermal treatment around 80-100°C, therefore, sugar degradation products is expected to be formed. Additionally, sugar degradation products may be formed during storage and this is known to promote anthocyanins degradation and also may reduce the stabilizing effect on color caused by decreasing of water activity [18].

In addition to the formation of polymeric pigments during processing, PC (%) markedly increased during storage and this fact plays an important role on the color stabilization. At a closer look to the Figure 3 it can be noticed that by occurrence of polymerized anthocyanins during storage, only minor changes were found for CD. This fact proves that the color provided by polymeric pigments compensates for a part of the color that was lost due to the extensive degradation of TMA during jam storage. In agreement with the results of other studies [5,34], the polymeric pigments formed in response to storage represent an important part of “stable color”. At the end of storage, the lowest values of PC (%), in the range 21-29%, were noticed in samples with LMAP and the highest, in the range 31-45%, for jams with HMP. Thus, as presented in Figure 3b, the lowest formation of polymeric pigments was observed in the jams prepared with LMAP to a level of 1%. Among the jams obtained with the same type of pectin, the increase in PC (%) during storage has been dose-dependent.

The star chart from Figure 2c shows the variation of PC (%) during storage. It can be seen that the highest value of PC (%) corresponds to HMP9.6 and the lowest to LMAP4.0. It was noticed an obvious link between the increasing of PC (%) and decreasing of TMA content during storage, (Figures 2c and 1a). It can be explain that the increases recorded in PC (%) throughout storage are due to the gradual inclusion of anthocyanins in polymeric pigments matrix. This finding is in line to those reported by Brownmiller *et al*. [15], Hager *et al*. [5] and Poiana *et al*. [26]. Our data revealed the protective effect of pectin on color quality during long-term storage of blackberry jam. The best stabilization of jam color during 6 months of storage was achieved by LMAP followed by LMP and HMP. It seems that the cross links formed in response to anthocyanins polymerization or condensation reactions among anthocyanins and procyanidins are no more stable than those occurring between anthocyanins and pectin.

### Evaluation of total phenolics content

In Table 3 are presented the changes recorded in TP content of jam samples in response to storage. Based on TP content found in blackberry jam one day post-processing and considering the amount of fruit required to obtain 100 g jam, it was possible to estimate the losses registered in response to processing. Other studies focused on this topic have proved that the losses registered in TP content as effect of thermal processing of various kinds of berries depend on the processing conditions, quality of fresh fruit as well as the jam formulation [7,8,24]. The thermal treatment applied for jam processing induced significant depreciations in TP content of jam samples obtained with LMAP, LMA and HMP as follows: 38-46%, 33-43% and 47-55% reported to the values recorded for corresponding fresh fruit. The most pronounced losses were noticed in blackberry jam with HMP and the lowest in samples obtained with LMAP. Therefore, by choosing of pectin with low DE and amidated groups, the retention of total phenolic compounds in jam could be improved. Also, by increasing of pectin dose from 0.3 to 1%, were noticed increases in TP content retained in jam samples analyzed in the range 14-17%, depending on the pectin type.

**Table 3:** The impact of storage at 20°C on TP content of blackberry jam

**Jam samples**	**TP (mg GAE·100**^**-1**^ **g jam)**			
	**1 day (0)**	**1 month**	**3 months**	**6 months**
LMP1	219.5 ± 9.8	204.1 ± 9.0^ns^	182.3 ± 7.2^*^	144.7 ± 7.3^***^
LMP2	201.2 ± 10.8	181.0 ± 10.3^ns^	162.6 ± 9.1^*^	126.4 ± 7.3^***^
LMP3	192.1 ± 10.7	164.1 ± 8.5^*^	141.3 ± 8.4^*^	104.8 ± 7.0^***^
LMAP4	237.2 ± 8.9	225.1 ± 9.8^ns^	205.6 ± 11.1^*^	169.3 ± 8.0^***^
LMAP5	224.3 ± 10.9	207.3 ± 11.3^ns^	189.2 ± 10.3^*^	152.2 ± 8.8^***^
LMAP6	203.2 ± 13.3	180.1 ± 11.1^ns^	160.3 ± 8.7^*^	121.6 ± 7.5^***^
HMP7	187.3 ± 11.3	166.5 ± 10.5^ns^	136.2 ± 7.2^**^	109.8 ± 6.0^***^
HMP8	175.4 ± 10.6	151.2 ± 7.4^*^	124.8 ± 7.4^**^	95.5 ± 6.1^***^
HMP9	160.1 ± 10.7	130.1 ± 5.8^*^	110.1 ± 6.3^**^	78.1 ± 5.6^***^

Statistical differences are indicated as follows: ns – non-significant, P > 0.1; * – significant, P < 0.05;

** – highly significant, P < 0.01 and *** – extremely significant, P < 0.001.

Legend: LMP1: LMP 1%; LMP2: LMP 0.7%; LMP3: LMP 0.3%; LMAP4: LMAP 1%; LMAP5: LMAP 0.7%; LMAP6: LMAP 0.3%; HMP7: HMP 1%; HMP8: HMP 0.7% and HMP9: HMP 0.3%.

The effect of jam storage on TP content is shown in Figure 1b. At the end of storage, the highest relative loss in TP content (51%) were recorded in jam sample with HMP to a dose of 0.3% and the lowest (29%) was noticed in jam with LMAP to a level of 1%. These findings revealed that the highest stability of TP throughout jam storage was achieved by LMAP, followed by LMP and HMP. Also, it was proved that the highest TP content in jam samples was provided by the largest dose of pectin.

**Figure 4:**
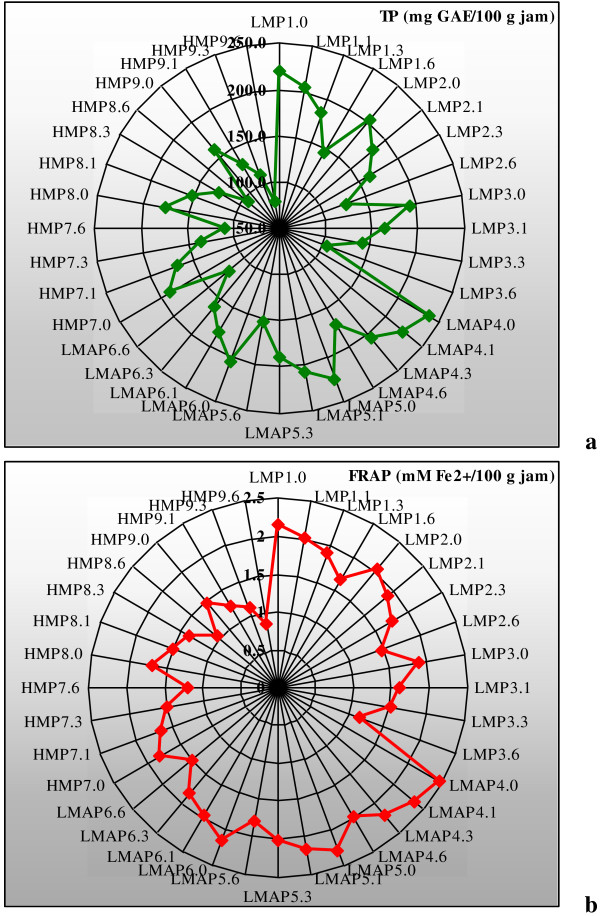
**Star representation of TP (a) and FRAP (b) variation during jam storage.** Legend: LMP1: LMP 1%; LMP2: LMP 0.7%; LMP3: LMP 0.3%; LMAP4: LMAP 1%; LMAP5: LMAP 0.7%; LMAP6: LMAP 0.3%; HMP7: HMP 1%; HMP8: HMP 0.7% and HMP9: HMP 0.3%. In samples labeled as LMP1.0, LMP1.6 and so on, the number after point represents the storage time.

Star chart representation of TP variation during storage is shown in Figure 4a. This chart highlights that the highest value of TP it was found in sample LMAP4.0 and the lowest for HMP9.6. The results of statistical processing by ANOVA test revealed that during jam storage, the significance of recorded changes varied with pectin type and its dosage. After 1 month of storage the differences reported to the control were quantified as non-significant (P > 0.1) and significant (P < 0.05), after 3 months the changes were significant (P < 0.05) and highly significant (P < 0.01), while over 6 months of storage the recorded differences have became extremely significant P < 0.001) for all jam samples.

### Evaluation of antioxidant activity

Based on the FPAP values recorded in fresh fruit and jam one day post-processing, Table 4, it can be estimated the losses in response to thermal processing. Therefore, it may be noticed that in jam with LMAP, LMA and HMP, the losses recorded in FRAP values were in the ranges: 28-37%, 18-28%, 40-52% of the value recorded for corresponding fresh fruit. Our results are close to those previously reported by other studies [5,6,13] and could be attributed to breakdown of polyphenols or any other biologically active compounds which are relatively unstable to thermal treatment. The best retention of antioxidant activity was registered in jam samples obtained with LMAP. Also, the FRAP values recorded in jam have been dependent on the pectin dose. By increasing of pectin dose from 0.3 to 1%, were recorded increases in FRAP values in the range 15-24%.

**Table 4:** The impact of storage at 20°C on FRAP values of blackberry jam

**Jam samples**	**FRAP (mM Fe**^**2+**^**·100**^**-1**^ **g jam)**			
	**1 day (0)**	**1 month**	**3 months**	**6 months**
LMP1	2.2 ± 0.2	2.0 ± 0.1^ns^	1.9 ± 0.1^ns^	1.6 ± 0.1^**^
LMP2	2.1 ± 0.1	1.9 ± 0.1^ns^	1.7 ± 0.1^*^	1.5 ± 0.1^**^
LMP3	1.9 ± 0.6	1.6 ± 0.1^ns^	1.5 ± 0.1^*^	1.2 ± 0.1^***^
LMAP4	2.5 ± 0.2	2.4 ± 0.2^ns^	2.1 ± 0.2^ns^	2.0 ± 0.2^*^
LMAP5	2.3 ± 0.2	2.2 ± 0.2^ns^	2.0 ± 0.2^ns^	1.8 ± 0.1^*^
LMAP6	2.1 ± 0.2	1.9 ± 0.2^ns^	1.8 ± 0.1^ns^	1.5 ± 0.1^**^
HMP7	1.8 ± 0.1	1.6 ± 0.1^ns^	1.5 ± 0.1^*^	1.2 ± 0.1^**^
HMP8	1.7 ± 0.2	1.5 ± 0.1^ns^	1.4 ± 0.1^*^	1.1 ± 0.1^***^
HMP9	1.5 ± 0.1	1.2 ± 0.1^ns^	1.1 ± 0.1^*^	0.9 ± 0.1^***^

Statistical differences are indicated as follows: ns – non-significant, P > 0.1; * – significant, P < 0.05;

** – highly significant, P < 0.01 and *** – extremely significant, P < 0.001.

Legend: LMP1: LMP 1%; LMP2: LMP 0.7%; LMP3: LMP 0.3%; LMAP4: LMAP 1%; LMAP5: LMAP 0.7%; LMAP6: LMAP 0.3%; HMP7: HMP 1%; HMP8: HMP 0.7% and HMP9: HMP 0.3%.

Figure 1c provides information regarding the relative losses recorded in FRAP values in response to storage. At the end of storage, the lowest relative losses in FRAP values, in the range 20-34%, were noticed in samples with 1% pectin and the highest (31-41%) in jam samples with 0.3% pectin decreases. Also, the highest FRAP values were registered in jams with LMAP followed by samples with LMP and HMP. Our data suggest that small changes in the composition of jam matrix, such as pectin type or its dosage, could affect the antioxidant properties of jam, probably due to the changes occurred in the interactions between food matrix ingredients.

From statistical analysis by ANOVA test it could be noticed that after 1 month of storage there were no statistical significant differences (p > 0.1) in FRAP values. After 3 months, the differences were non-significant (p > 0.1) for samples with LMAP and the both, non-significant (p > 0.1) and significant (P < 0.05) for samples with LMP and HMP.

From chart presented in Figure 4b it can be seen the FPAP variation in response to jam storage. The highest FRAP value corresponds to jam sample LMAP4.0 and the lowest to HMP9.6. The sample LMAP4 followed by LMAP5 present the smallest losses of FRAP values over 6 months of storage. In terms of statistical analysis, these alterations were significant (P < 0.05) and highly significant (P < 0.01) for jam samples with LMAP and highly significant (P < 0.01) and extremely significant (P < 0.001) for those samples obtained with LMP or HMP. These findings suggest that antioxidant properties were best protected in samples with LMAP in response to long-term storage.

Total phenolic compounds but especially anthocyanin pigments greatly contribute to the antioxidant activity of blackberries and corresponding jams [2,5]. The studies carried out by Tsai *et al.*[35], Brownmiller *et al.*[15] and Hager *et al*. [5] highlighted that polymeric anthocyanins resulted in response to processing and storage exhibited antioxidant activity. Also, Kopjar *et al*. [18] mentioned that some degradation products of TMA resulting in response to thermal treatment displayed antioxidant activity. Our data revealed that after 6 months of storage, the losses recorded for FRAP values were lower than those registered for TP or TMA. Thus, polymeric pigments [34,35] and other compounds [5,8] formed during heating and storage could compensate for a part of the antioxidant activity that was lost due to the anthocyanins degradation. Although this study does not completely confirm the antioxidant properties of polymeric pigments, it can be used as a basis for further studies. These findings could be useful to optimize jam processing technology in order to improve the health promoting properties of these products.

From the star representations, Figures 2 and 4, it can be seen that the highest variation recorded in the storage time is given by TP followed by TMA and PC (%). Lower variation presented FRAP and CD.

**Figure 5:**
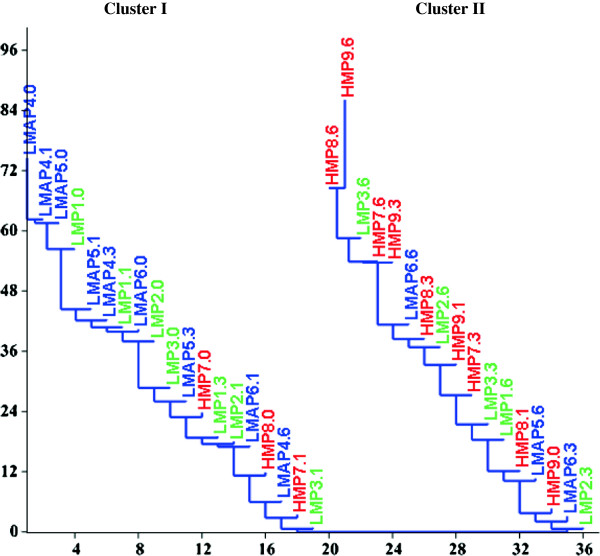
**Representation of neighbor-joining cluster analysis of jams based on TMA, TP and FRAP.** Legend: LMP1: LMP 1%; LMP2: LMP 0.7%; LMP3: LMP 0.3%; LMAP4: LMAP 1%; LMAP5: LMAP 0.7%; LMAP6: LMAP 0.3%; HMP7: HMP 1%; HMP8: HMP 0.7% and HMP9: HMP 0.3%. In samples labeled as LMP1.0, LMP1.6 and so on, the number after point represents the storage time. **Cluster I**: LMAP4.0 > LMAP5.0 > LMAP4.1 > LMP1.0 > LMAP5.1 > LMAP6.0 > LMAP4.3 > LMP1.1 > LMP2.0. >LMAP5.3 > LMP3.0 > LMAP6.1 > LMP2.1 > LMP1.3 > LMAP4.6 > LMAP6.3 > HMP7.0 > LMP3.1. **Cluster II**: LMP2.3 > HMP8.0 > LMAP5.6 > HMP7.1 > LMP3.3 > HMP9.0 > HMP8.1 > LMP1.6 > LMAP6.6>. HMP7.3 > LMP2.6 > HMP8.3 > HMP9.1 > LMP3.6 > HMP9.3 > HMP7.6 > HMP8.6 > HMP9.6.

From Neighbor-Joining Cluster analysis based on TMA, TP and FRAP, Figure 5, it can be noticed two clusters, one cluster joining the jam formulations that maintain the highest levels of antioxidant parameters (Cluster I) and the second cluster revealing the formulations with largest loss of desired antioxidant properties (Cluster II). Usually, this analysis is used as a clustering method for the creation of phenograms, but it can also be used as a classification method to identify the best methods from a set of multiple procedures involving multiple variables [36]. According to the Neighbor-Joining Cluster analysis, we can recommend the following types and doses of pectin related to the storage period for processing of blackberry jam with high levels of antioxidant parameters: LMAP 1% (0 to 6 months), LMAP 0.7% (1 to 3 months), LMP 1% (0 to 3 months), LMAP0.3% (0 to 3 months), LMP0.7% (0 to 1 month) and LMP0.3% (0 to 1 month).

#### *Experimental research*

**Berries** Blackberries (*Rubus fructicosus L.*) have been harvested in the summer of 2011 at the fully ripe stage from their natural habitat (mountainous regions of south-western Romania, altitude 1000–1200 m). The berries were stored in polyethylene bags and kept in refrigerated conditions (4-6°C) for 1 day until analysis and jam processing.

### Pectin

The following commercial pectins purchased from Danisco Ingredients (Denmark) were used in this study: Grindsted Pectin SS 200, Grindsted Pectin LC 950 and Grindsted Pectin LA 410. Grindsted Pectin SS 200 is a slow-set high-methoxyl pectin (HMP), calcium insensitive with DE 65% and DA 0%. It is a powder manufactured from citrus peel and has natural color variation from off-white to golden. Grindsted Pectin LC 950 is a low-methoxyl pectin (LMP), high-calcium reactive with DE 30%, non-amidated or conventional pectin with DA 0%. It is a powder manufactured from citrus peel and has natural color variation from off-white to golden. Grindsted Pectin LA 410 is a low-methoxyl amidated pectin (LMAP), high-calcium reactive with DE 30% and DA 20%. It is an off-white powder manufactured from citrus peel. Prior to use, pectins were dispersed with deionised water using a blender MOULINEX AAW445 for homogenization.

### Jam processing

Blackberry jam processing has been performed in laboratory conditions, under atmospheric pressure, according to a traditional procedure used in households or small-scale systems. Firstly, fruit purée was prepared by blending of blackberry fruit (1500 g). Then, fruit purée with 783 g sucrose was stirred and heated to 90°C in a double-walled vessel from stainless steel. The pectins were separately added under continuous stirring at the final stage of the jam cooking in order to assure three levels of concentrations, 0.3, 0.7 and 1.0% (m/m), respectively. The mixtures were adjusted to pH 2.90 ± 0.05 (for jams with HMP) and 3.3 ± 0.05 (for jams with LMAP and LMP) with citric acid (400 g/L). Additionally, calcium chloride dihydrate (CaCl_2_ ∙ 2H_2_O) was added in jam formulations with LMP and LMAP to a concentration of 20 mg calcium ions**/**g pectin for Grindsted Pectin LA 410, respectively 40 mg calcium ions/g pectin for Grindsted Pectin LC 950. The calcium ions dose/g pectin was established according to manufacturer's recommendations depending on the each type of pectin. Time of cooking was approximately 20 minutes at 90°C followed by heating to full boiling (101-103°C) for 2–3 minutes. When the final TSS of mixture reached 45°Bx, the obtained mass were cooled down to 80°C and hot-packed into 100 g transparent glass jars with screw caps and then pasteurized at 80°C for 10 min according to the procedure described by Kovacevic *et al*. [37]. Further, the jams allowed to cool at room temperature and kept at 20°C until analysis, in a storeroom with low lightness, without direct exposure to sunlight, similar to those used in households to store canned fruits. The weight of the jam before and after cooking was performed. The difference in weight represents the amount of water lost during processing (approximately 34 g for 1 kg jam). The jam samples were analyzed one day post-processing and after 1, 3 and 6 months of storage for TMA, CD, PC (%), TP and FRAP values.

### Analytical procedures

#### *Total soluble solids*

TSS of fruit and jam, expressed as degree Brix (°Brix), was determined at 20°C by refractometry using a digital handheld refractometer DR301-95 (KRÜSS, Germany) according to standard method as described by AOAC [38].

#### *Total phenolic assay*

TP content was quantified according to the method described by Singleton *et al.*[39]. The extracts for TP analysis were obtained from fresh fruits and related jams according to Kalt *et al.*[40] by mixing of crushed samples (~5 g) with 10 mL hot ethanol 95% (v/v)) for 2 min. Then, the mixture was filtered and the residue was re-extracted twice by the same procedure. The obtained extracts were combined. In agreement to this protocol, an aliquot of 0.5 mL extract previously diluted 1:100 (v/v) with distilled water was mixed with 2.5 mL of Folin-Ciocalteau reagent (previously diluted 1:10 with distilled water). After 3 min, 2 mL of 7.5% of sodium carbonate was added and the contents were mixed thoroughly. The color was developed and absorbance was measured at 750 nm using a UV–VIS spectrophotometer (Analytic Jena SPECORD 205) after 60 min using gallic acid as a standard. Results were quantified as mg GAE per 100 g fresh fruit, respectively jam (when were analyzed TP for jam).

#### *Antioxidant activity (FRAP assay)*

Antioxidant activity of samples were measured according to the FRAP assay (ferric reducing antioxidant power) as described by Benzie and Strain [41]. By this colorimetric test it can be measured the ability of samples to reduce the ferric 2,4,6-tris(2-pyridyl)-1,3,5-triazine (TPTZ) complex to a blue-colored ferrous form at 37°C in pH 3.6 sodium acetate buffer, accompanied by its absorbance change. The extracts for FRAP assay were obtained from fruits and jams according to Kalt *et al.*[40] by mixing of crushed samples (~20 g) with 20 mL ethanol 95% (v/v) acidified with HCl (0.1%, v/v) for 60 min followed by the filtering of solution. The residue was re-extracted twice and the obtained extracts were combined and diluted to the volume of 50 mL with ethanol acidified with HCl (0.1%). An aliquot of 0.5 mL extract previously diluted 1:100 (v/v) with distilled water was mixed with FRAP reagent and the samples measured after 30 min at 593 nm using FeSO_4_ · 7H_2_O as a standard. The antioxidant activity was expressed as mM Fe^2+^ equivalents per 100 g fresh fruit, respectively jam.

#### *The monomeric anthocyanin pigment content and color indices*

TMA content of samples was determined using the pH-differential method [33]. The extracts were prepared according to Kalt *et al.*[40] by mixing of crushed fruit or jam (~5 g) with 20 mL ethanol 95% (v/v) acidified with HCl (0.1%, v/v) for 2 min. The obtained mixture was kept at room temperature in the dark for 16 h and then filtered using Whatman No. 3 filter paper. The resulted extracts were diluted 15-fold with 0.025 M potassium chloride buffer (pH = 1.0) or 0.4 M sodium acetate buffer (pH = 4.5). A UV-visible spectrophotometer (SPECORD 205 by Analytik Jena) and 1-cm path length disposable glass cells were used for spectral measurements at 520 and 700 nm against distilled water as blank. Pigment content was calculated as milligrams cyanidin-3-glucoside per 100 g fresh fruit or jam using a molar extinction coefficient of 26 900 L/cm/mol and molecular weight of cyanidin-3-glucoside (449.2 g/mol). CD, PC and PC (%) were determined using the bisulfite bleaching method as described by Giusti and Wrolstad [33].

#### *Statistical analysis*

All analyses were carried out in triplicates and results were expressed as means ± standard deviation. Statistical data processing was performed by one-way analysis of variance (ANOVA) using Statistical Analysis System, SAS (Software Version 8.1; SAS Institute, Inc., Cary, NC) [42]. Computations Tukey post-hoc means comparisons and Levene’s test for equal variance was also used to assess the difference between group means. To represent the variation of the studied parameters in storage time we have used star charts which plot the values of each category along a separate axis that starts in the center of the chart and ends on the outer ring. Star charts are a useful way to display multivariate observations with an arbitrary number of variables [43]. Neighbor-Joining Cluster analysis was performed by using Past Software Packages [44] for clustering of jam samples based on TMA, TP and FRAP in order to identify the best methods for blackberry jam processing. Neighbor-Joining clustering is an alternative method for hierarchical cluster analysis [36], quite truthful, for small data sets [45].

## Conclusion

The present study demonstrates that both thermal processing and jam storage resulted in significant losses of TMA, TP, FRAP values and color indices but the extent of recorded losses was closely related to the pectin type and dosage. It was noted an obvious connection between the increasing of PC (%) and the decreasing of TMA due to their gradual inclusion in polymeric pigments matrix during jam storage. The pectin type strongly affected the anthocyanins stability during storage. Thus, the retention degree of these compounds was higher in jams with low-esterified pectins than in samples with high-esterified pectin. In jams with low-esterified pectin, anthocyanins stabilization was better in those samples obtained with pectin having similar DE and amidation, respectively. Over 6 months of storage, the pigments and antioxidant properties were best retained in the jams obtained with LMAP, followed by samples with LMP and HMP. Moreover, the amounts of biologically active compounds retained in jam one day post-processing have been dependent on the pectin dosage. Thus, jam formulation is very important considering that the composition of the matrix strongly affects its antioxidant properties due to the changes occurred in interactions between matrix constituents. Small changes in the jam matrix composition, such as pectin type or its dosage, greatly affect the jam quality. Our results suggest that by a proper selection of pectin type and dose in the formulation could be improve the degree of bioactive compounds retention in the gelled products, reducing in this way the losses recorded in response to thermal processing and storage. We can conclude that LMAP to a level of 1% is the most indicated for processing of blackberry jam with the highest antioxidant properties and color stability.

## Abbreviations

HMP: High-methoxyl pectin; LMP: Low-methoxyl pectin; LMAP: Low-methoxyl amidated pectin; TMA: Total monomeric anthocyanins; DE: Degree of esterification; DA: Degree of amidation; TP: Total phenolics; FRAP: Ferric reducing antioxidant power; GAE: Gallic acid equivalent; CD: Color density; PC: Polymeric color; PC (%): Percent of polymeric color; Fw: Fresh weight; TSS: Total soluble solids.

## Competing interests

The authors declare no conflict of interest.

## Authors’ contributions

MAP performed jam processing, TMA and color analysis, results interpretation and manuscript preparation. MFM performed extractions, TP analysis and contributed to data interpretation and manuscript preparation. DMB contributed to the color and FRAP analysis, performed statistical analysis and helped to data interpretation. RG contributed to FRAP analysis, help to statistical processing and data interpretation. EA contributed to jam processing, color analysis and data interpretation. All authors read and approved the final version of the manuscript.
